# Factors affecting vitamin D status in different populations in the city of São Paulo, Brazil: the São PAulo vitamin D Evaluation Study (SPADES)

**DOI:** 10.1186/1472-6823-13-14

**Published:** 2013-04-29

**Authors:** Sergio Setsuo Maeda, Gabriela Luporini Saraiva, Ilda Sizue Kunii, Lilian Fukusima Hayashi, Maysa Seabra Cendoroglo, Luiz Roberto Ramos, Marise Lazaretti-Castro

**Affiliations:** 1 Department of Medicine, Division of Endocrinology, Universidade Federal de São Paulo (UNIFESP) – Escola Paulista de Medicina, São Paulo, São Paulo, Brazil; 2 Department of Medicine, Geriatrics, UNIFESP, São Paulo, São Paulo, Brazil; 3 Department of Preventive Medicine, UNIFESP, São Paulo, São Paulo, Brazil

**Keywords:** 25 hydroxyvitamin D, Parathyroid Hormone, Seasonal influence, Ultraviolet radiation

## Abstract

**Background:**

Hypovitaminosis D is a common condition among elderly individuals in temperate-climate countries, with a clear seasonal variation on 25 hydroxyvitamin D [(25(OH)D] levels, increasing after summer and decreasing after winter, but there are few data from sunny countries such as Brazil. We aimed to evaluate 25-hydroxyvitamin D concentrations and its determining factors, in individuals in the city of São Paulo belonging to different age groups and presenting different sun exposure habits.

**Methods:**

591 people were included as follows: 177 were living in institutions (NURSING HOMES, NH, 76.2 ± 9.0 years), 243 were individuals from the community (COMMUNITY DWELLINGS, CD, 79.6 ± 5.3 years), 99 were enrolled in physical activity program designed for the elderly (PHYSICAL ACTIVITY, PA, 67.6 ± 5.4 years) and 72 were young (YOUNG, 23.9 ± 2.8 years). Ionized calcium, PTH, 25(OH)D, creatinine and albumin were evaluated. ANOVA, Mann–Whitney and Kruskal Wallis tests, Pearson Linear Correlation and Multiple Regression were used in the statistical analysis.

**Results:**

25(OH)D mean values during winter for the different groups were 36.1 ± 21.2 nmol/L (NH), 44.1 ± 24.0 nmol/L (CD), 78.9 ± 30.9 nmol/L (PA) and 69.6 ± 26.2 nmol/L (YOUNG) (p < 0.001) while during summer they were 42.1 ± 25.9 nmol/L, 59.1 ± 29.6 nmol/L, 91.6 ± 31.7 nmol/L and 103.6 ± 29.3 nmol/L, respectively (p < 0.001). The equation which predicts PTH values based on 25(OH)D concentration is PTH = 10 + 104.24.e^-(vitD-12.5)/62.36^ and the 25(OH)D value above which correlation with PTH is lost is 75.0 nmol/L. In a multiple regression analysis having 25(OH)D concentration as the depending variable, the determining factors were PTH, ionized calcium and month of the year (p < 0.05).

**Conclusions:**

Much lower 25(OH)D values were found for the older individuals when compared to younger individuals. This finding is possibly due to age and habit-related differences in sunlight exposure. The existence of seasonal effects on 25(OH)D concentration throughout the year was evident for all the groups studied, except for the nursing home group. According to our data, PTH values tend to plateau above 75 nmol/L.

## Background

Hypovitaminosis D is well documented, mainly among elderly populations in countries from the northern hemisphere [1-4]. For the elderly, hypovitaminosis D stimulates the parathyroid gland, both directly and indirectly, leading to secondary hyperparathyroidism, bringing consequences such as bone mass loss and an increased risk for fractures [3]. For the young population this correlation is not clear. The concentrations of 25-hydroxyvitamin D [25(OH)D] are influenced by several factors, such as the age, skin tone, latitude, time of the day, season, the weather and many others. However, some authors evidenced that, different from what is expected, people from southern European countries have lower concentrations of 25(OH)D than people from northern countries [1,5]. These data suggest that habit-related factors also influence 25(OH)D concentrations and these have not been investigated in countries where sunlight is abundant, such as Brazil.

This study was named SPADES (The São PAulo Vitamin DEvaluation Study) and its main objective was to recognize the factors that contribute to determining blood 25(OH)D concentrations in four different population groups in the city of São Paulo, Brazil (23°34’S, subtropical weather).

## Methods

### Subjects

The study protocol was previously approved by the UNIFESP Ethics Committee and all volunteers gave written informed consent.

The cross-sectional study SPADES aimed at analyzing data from different age groups in the population and also groups that have distinct sunlight exposure habits in the city of São Paulo. All the individual population groups reported here have already had their results published elsewhere and the objective of the present study is to evaluate all the groups together. The data were obtained in the same city and the same method was used for measuring 25(OH)D [6-10].

The first population studied was called NURSING HOMES (NH) and it was composed of individuals who live in two nursing homes in the city of São Paulo. Mostly these individuals are from low income families that have little access to health services and for this reason their health conditions were generally poor. Individuals showing creatinine values above 2.0 mg/dL, hypercalcemia, hypocalcemia and those who were confined to a bed were excluded from the study. The final sample was represented by 177 individuals, ages varying from 60 to 100 years. Ethnic distribution: 90.9% white and 9.1% black. Blood samples were drawn in April (fall, 37.3% of individuals) and July (winter, 62.7% of individuals) of 2001 [6,7].

The second population was called COMMUNITY DWELLINGS (CD) and it originated from a cohort study (EPIDOSO) developed with people from the district around UNIFESP (Universidade Federal de São Paulo), one of the 55 districts that compose the city of São Paulo. This region is predominantly composed by a Caucasian middle class population. We analyzed the data from 250 individuals, evaluated in 2001, ten years after the beginning of the study. From those, seven were excluded from the study (one for showing creatinine levels above 2.0 mg/dL and six for showing hypercalcemia). The final population studied was composed by 243 individuals, ages varying from 61 to 97 years [6-8]. Ethnic distribution: 94.6% white and 5.4% black. In this group, 35.0% of the individuals had their blood samples collected during summer/fall, while 65.0% had them collected during winter/spring.

The third population was called PHYSICAL ACTIVITY (PA) and was composed of 101 individuals (54 women and 47 men), enrolled in a physical activity program destined to the elderly [9]. Each volunteer had a blood sample collected in two different moments in 2002: one in June (winter) and one in December (summer). Two patients were excluded from the initial group: one for presenting primary hyperparathyroidism (PTH 163 pg/mL and ionized calcium 1.56 mM) and the other for presenting 25(OH)D level much higher than normal (280 nmol/L). The final population studied was composed of 99 people and from those, 88 had the second blood sample collected (88.9% of the initial population). Ages varied from 55 to 83 years. Ethnic distribution: 60.6% white, 19.2% Asian, 12.1% black and 8.1% native.

The fourth population was designated YOUNG and was composed of 72 individuals, age varying from 17 to 35 years. Ethnic distribution: 75% white, 19.4% Asian and 5.6% black. The blood samples were obtained between August 2002 and February 2003 [10]. In this group, 51.4% of the individuals had their blood sample collected in months corresponding to summer/fall and 48.6% in months corresponding to winter/spring.

The final population of the SPADES study was composed of 591 individuals.

## Methods

The blood samples were taken after an eight-hour fast. Ionized calcium, creatinine, albumin, 25(OH)D and intact parathyroid hormone (PTH) were measured. All serum samples were collected into refrigerated tubes, processed in refrigerated centrifuges, and frozen at −20°C until measured. The ionized calcium was measured after centrifugation.

Creatinine and albumin were measured by an automatic colorimetric method (Modular Roche, São Paulo, Brazil). The ionized calcium was measured using a specific ion-electrode method (AVL 9180 Electrolyte Analyzer, Minn). PTH (parathyroid hormone) was measured by an immunofluorimetric in house method (IFMA, VR: 10–55 pg/mL) [11]. The creatinine clearance was calculated by the Cockroft-Gault formula [12]. Reference values for these methods are presented in Table 1.

**Table 1:** Mean ± standard deviation and reference values (RV) for the variables studied and its comparison, according to the gender and group of origin

	**NH**	**CD**	**PA**	**Young**
	**(N = 177)**	**(N = 243)**	**(N = 99)**	**(N = 72)**
	**M = 49 W = 128**	**M = 75 W = 168**	**M = 47 W = 52**	**M = 32 W = 40**
**Age (years)**	76.2 ± 9.0	79.6 ± 5.3*^b^	67.6 ± 5.4* ^**Ŧ**a^	23.9 ± 2.8*^Ŧb^
Male	73.8 ± 8.8	79.4 ± 4.4*^ab^	67.8 ± 4.9*^Ŧa^	23.8 ± 2.7*^Ŧb^
Female	77.7 ± 8.9	79.7 ± 5.6^a^	67.4 ± 5.9*^Ŧa^	23.9 ± 2.9*^Ŧb^
**BMI (RV: 19.0-25.0 kg/m**^**2**^**)**	26.3 ± 5.6	26.7 ± 4.3	27.2 ± 4.4^a^	22.4 ± 3.0*^Ŧ^
Male	25.2 ± 4.9	25.8 ± 3.4^£a^	28.1 ± 4.0^£a^	23.1 ± 2.3*^£^
Female	26.8 ± 5.9	27.2 ± 4.6^£a^	27.6 ± 4.7^£a^	21.9 ± 3.4^£^*^Ŧ^
**Albumin (RV: 3.5-5.0 g/L)**	3.7 ± 0.4	4.0 ± 0.3*	4.0 ± 0.2*^Ŧ^	4.7 ± 0.5*^Ŧb^
Male	3.6 ± 0.4	4.1 ± 0.2*^a^	4.1 ± 0.2*^a^	5.0 ± 0.3^£Ŧ^*
Female	3.7 ± 0.4	4.0 ± 0.3*^a^	4.0 ± 0.2*^a^	4.5 ± 0.4^£Ŧ^*
**Creatinine (RV: 0.6-1.5 mg/dL)**	1.04 ± 0.3	0.95 ± 0.2*^a^	0.98 ± 0.1	1.07 ± 0.2
Male	1.15 ± 0.2^£^	1.16 ± 0.2^£^	1.07 ± 0.1^£a^	1.20 ± 0.1^£^
Female	1.00 ± 0.2^£^	0.85 ± 0.1^£^*	0.89 ± 0.1^£^*	0.96 ± 0.1^£**Ŧ**^
**ClearCrea (RV: 70.0-110.0 mg/dL)**	49.7 ± 23.2	53.8 ± 15.2	68.5 ± 19.7*^Ŧa^	88.5 ± 20.8*^Ŧ^
Male	56.6 ± 23.2^£^	54.6 ± 17.0^£^	70.2 ± 17.7*^Ŧa^	96.5 ± 13.8^£^*^Ŧ^
Female	47.1 ± 22.7^£^	48.5 ± 23.3^£^	66.9 ± 21.5*^Ŧa^	82.2 ± 23.3^£^*^Ŧ^
**Ionized Calcium (RV: 1.12-1.40 mM)**	1.28 ± 0.04	1.25 ± 0.05*	1.31 ± 0.04*^Ŧ^	1.31 ± 0.05*^Ŧ^
Male	1.28 ± 0.10	1.26 ± 0.10*	1.31 ± 0.03*^Ŧ^	1.33 ± 0.10^£^*^Ŧ^
Female	1.29 ± 0.10	1.25 ± 0.10*	1.32 ± 0.04*^Ŧ^	1.29 ± 0.10^£^*^Ŧ^
**PTH (RV: 10.0-55.0 pg/mL)**^**#**^	85.0 ± 55.8	73.8 ± 56.6^ab^	25.0 ± 12.3*^Ŧ^	26.4 ± 15.4*^Ŧ^
Male	85.0 ± 58.2	78.0 ± 81.7	26.1 ± 15.7*^Ŧ^	26.8 ± 17.8*^Ŧ^
Female	84.8 ± 55.1	71.8 ± 39.8*	23.6 ± 9.5*^Ŧ^	26.1 ± 13.2*^Ŧ^
**25(OH)D (RV: >75.0 nmol/L)**^**#**^	37.6 ± 29.9	49.5 ± 27.7*^ab^	78.9 ± 30.9*^Ŧ^	86.3 ± 34.8*^Ŧ^
Male	47.1 ± 20.8^£^	59.4 ± 30.9^£^	81.2 ± 30.1*^Ŧ^	94.3 ± 38.2*^Ŧ^
Female	33.9 ± 32.1^£^	45.1 ± 25.1^£^*	76.7 ± 31.8*^Ŧ^	79.9 ± 30.9*^Ŧ^

*M* = men *W* = women.

^£^ significant difference (p < 0.05) in relation to gender inside each group.

* significant difference (p < 0.05) in relation to NURSING HOMES (NH).

^**Ŧ**^ significant difference (p < 0.05) in relation to COMMUNITY DWELLINGS (CD).

^a^ significant difference (p < 0.05) in relation to YOUNG .

^b^ significant difference (p < 0.05) in relation to PHYSICAL ACTIVITY (PA).

# Non-parametric analysis (Kruskal-Wallis e Wilcoxon tests).

Other variables (One-Way Anova and Scheffé Post Hoc).

The 25(OH)D concentrations were determined by an immunoradiometric assay (Nichols Institute Diagnostics, San Juan Capristrano, CA, USA). Intra-assay coefficient of variation was 4.8%, and inter-assay coefficient of variation was 16.0% for the lowest values (mean: 35.5 nmol/L) and 3.0% for the highest control (mean: 154.0 nmol/L).

To certify that the radiation conditions were similar through the years, we compared ultraviolet radiation during 4 consecutive years, and the UV year-pattern was repetitive (p = 0,444, unpublished data).

**Figure 1:**
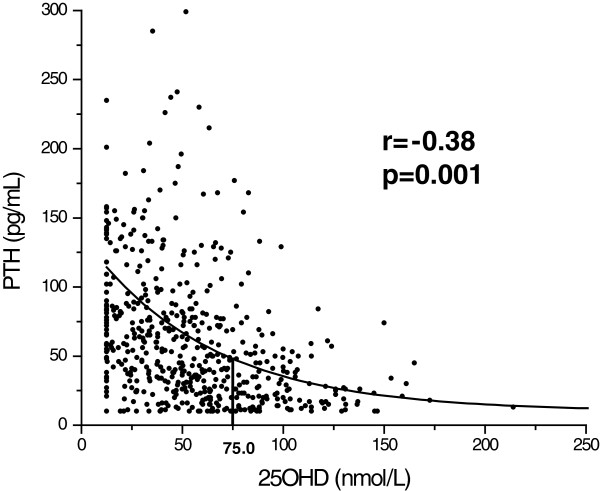
**Correlation between PTH (pg/mL) and 25OHD (nmol/L) in the sample studied.** Above 75.0 nmol/L of 25OHD there is no correlation with PTH.

The exponential formula that predicts PTH values from 25(OH)D was created using the software Origin 5.0 (Microcal Inc, Northampton, Massachusetts, USA). This same program was used for generating Figure 1.

### Statistical analysis

The Mann–Whitney test was used to evaluate the differences between men and women, and ANOVA and Kruskal Wallis test were used to check for the difference between the groups according to their origin. The correlation between 25(OH)D and PTH with the other quantitative variables was evaluated through the Pearson Linear Correlation and Multiple Regression. Data are reported as Mean ± SD (Standard Deviation). Differences were considered significant when p < 0.05.

## Results

A total of 591 individuals were included as follows: NH, 177 individuals (128 women and 49 men, mean age 76.2 ± 9.0 years); CD, 243 individuals (168 women and 75 men, mean age 79.6 ± 5.3 years); PA, 99 individuals (52 women and 47 men, mean age 67.6 ± 5.4 years) and YOUNG, 72 individuals (40 women and 32 men, mean age 23.9 ± 2.8 years). In Tables 1, 2 and 3, only 25(OH)D measurements done during winter were used for the group PA. In relation to ethnicity, the sample was predominantly composed of whites (84.4%).

**Table 2:** Correlation between PTH (pg/mL) and 25(OH)D (nmol/L) considering different 25(OH)D concentration ranges

**25(OH)D (nmol/L)**	**n**	**r**	**p**	**PTH (pg/mL)**
All	572	−0.330	**>0.001**	62.6 ± 53.6
>25.0	460	−0.303	**>0.001**	58.0 ± 54.3
>37.5	373	−0.271	**>0.001**	51.9 ± 44.3
>50.0	292	−0.236	**>0.001**	47.2 ± 39.6
>62.5	205	−0.193	**0.006**	43.0 ± 35.6
>75.0	139	−0.137	0.114	39.4 ± 31.6
>87.5	93	−0.062	0.558	37.4 ± 26.5
>100.0	59	+0.138	0.306	36.4 ± 25.0

**Table 3:** 25(OH)D (nmol/L) and PTH (pg/mL) concentrations according to the different age groups (vitamin D users were excluded), percentage of individuals with secondary hyperparathyroidism (SHP) within each group and correlation between PTH and 25(OH)D evaluated by Pearson’s correlation

**Age Group (years)**	**n**	**25(OH)D (nmol/L)**	**< 75.0 nmol/L (%)**	**PTH (pg/mL)**	**SHP (%)**	**r**	**p**
17–35	72	86.3 ± 34.9	40.3	26.4 ± 15.4	6.9	−0.018	0.876
55–60	13	62.9 ± 35.7	64.2	50.8 ± 44.5	28.5	−0.185	0.546
61–70	97	60.9 ± 34.6	50.0	48.7 ± 46.3	25.0	−0.417	**< 0.001**
71–80	218	49.5 ± 28.4	84.8	68.3 ± 44.9	46.0	−0.309	**< 0.001**
81–90	132	43.8 ± 29.0	78.4	80.0 ± 71.2	52.5	−0.138	0.139
91–100	13	30.6 ± 17.2	100.0	123.4 ± 67.7	58.9	−0.398	0.225

The laboratory results obtained for the group as a total and also separated by gender and subgroup of origin are described in Table 1. Men presented higher creatinine, creatinine clearance and 25(OH)D values, when compared to women, but the sex ratio between groups differs significantly; there are more women in NH (72.3%) and CD (69.1%) groups than in the PA (52.5%) and YOUNG (55.5%) groups. We observed that people in YOUNG group had a lower BMI and higher albumin and creatinine clearance values when compared to the other groups.

Only 6.3% of the total population studied had made use of vitamin D supplements (dose varying from 200 to 400 IU a day) and these had a 25(OH)D average blood concentration of 69.1 ± 34.9 nmol/L, while the group that had not taken a vitamin D supplement showed a significantly lower blood concentration (54.8 ± 33.4 nmol/L, p = 0.018).

Using the Pearson´s linear correlation and considering only individuals who were not using vitamin D supplements, significant correlations were found between 25(OH)D and age (r = −0.43, p = 0.001), PTH (r = −0.38, p = 0.001), albumin (r = +0.32, p = 0.001), creatinine clearence (r = +0.31, p = 0.001), ionized calcium (r = +0.30, p = 0.001), month of the year (r = −0.29, p = 0.001) and BMI (r = −0.21, p = 0.001). Creatinine did not show any significant correlation (r = +0.04, p = 0.203). Using a model of multiple linear regression, setting the age as the predictive variable and adjusting by ethnicity and gender, the following correlations were still significant: PTH (p = 0.01), ionized calcium (p = 0.048) and month of the year (p = 0.01).

Considering the PTH values, significant correlations were found with the following variables: age (r = +0.37, p = 0.001), 25(OH)D (r = −0.38, p = 0.001), albumin (r = −0.30, p = 0.001), creatinine clearence (r = −0.29, p = 0.001), creatinine (r = +0.13, p = 0.003), ionized calcium (r = −0.25, p = 0.001) and BMI (r = −0.13, p = 0.003). Using a model of multiple linear regression, setting the age as the predictive variable, 25(OH)D (p = 0.001), creatinine (p = 0.001) and ionized calcium (p = 0.011) were still significant.

The mathematical equation which predicts the PTH value, based on the 25(OH)D concentration is expressed by the formula PTH = 10 + 104.24.e^-(vitD-12.5)/62.36^. We also defined the concentration of 75.0 nmol/L as the cut-off value for which 25(OH)D concentrations did not correlate with PTH levels (Figure 1, Table 2). Within the groups, 91.5% from NH; 87.6% from CD; 48.5% from PA and 40.3% from YOUNG fell below this concentration cut-off.

In Table 4 reports on the 25(OH)D concentration distribution (nmol/L) according to the group and season of the year. Summer and fall were grouped together, considering these are very sunny. We observed that the NH and CD groups were the ones showing the lowest 25(OH)D concentration throughout the year (36.1 ± 21.2 and 44.1 ± 24.0 nmol/L during winter/spring and 42.1 ± 25.9 and 59.1 ± 29.6 nmol/L during summer/fall, respectively). The group NH was the only one not to show any increase during summer/fall.

**Table 4:** Mean ± standard deviation referring to the comparisons of 25(OH)D according to the origin of the sample and season of the year

**Season of the year**				
		**Winter/Spring**		**Summer/Fall**
**Origin**	**n**	**25(OH)D (nmol/L)**	**n**	**25(OH)D (nmol/L)**
**NH**	111	36.1 ± 21.2	66	42.1 ± 25.9
**CD**	158	44.1 ± 24.0	85	59.1 ± 29.6 ^**t**^
**PA**^**£**^	99	78.9 ± 30.9 ^a b^	88	91.6 ± 31.7 ^**t** a b^
**Young**	35	69.3 ± 25.6 ^a b^	37	103.6 ± 29.3 ^**t** a b^

^£^ paired analysis.

^t^ significant difference (p < 0.01) within each origin, considering the season of the year.

^a^ significant difference (p < 0.05) within each season of the year in relation to NURSING.

^b^ significant difference (p < 0.05) within each season of the year in relation to COMMUNITY.

Within each age group, as age advances, progressively lower 25(OH)D and higher PTH concentrations were observed. 25OHD concentrations lower than 75.0 nmol/L were found for 85.7% of those older than 71 years, while the incidence of secondary hyperparathyroidism was 57% among those (Table 3).

## Discussion

Several reports have previously shown that the prevalence of hypovitaminosis D is high among the elderly in the United States and Europe (< 30 ng/mL), but there are few data from countries in South America, where sunlight is abundant throughout the year, such as Brazil [1-4]. The abundant sunlight would make us believe this was a problem restricted to countries of higher latitudes. However, Lips *et al.* and van der Wielen *et al.* demonstrated that countries from southern Europe show higher incidence of hypovitaminosis D when compared to northern countries [1-5]. Some recent reviews showed that the vitamin D deficiency is common in South Asia and the Middle East [13,14].

The city of São Paulo is crossed by the Tropic of Capricorn (23°S) and presents relatively mild winters, with temperatures rarely achieving 0°C (32°F). It is a large urban center, being the largest one in South America, with a predominant Caucasian population. It has more than 10 million inhabitants and a large concentration of high-rise buildings and industries. During winter there is a significant concentration of air pollutants which can absorb solar UVB radiation and, consequently, reducing vitamin D formation [15]. Our elderly population has been increased similar to the pattern of developed countries. Between 1980 and 2003, there was an increment of 8.7 years in the life expectancy at birth, which nowadays is 71.3 years in Brazil.

Age was the variable that most significantly correlated with 25(OH)D plasma concentrations, as was demonstrated by a multiple regression model applied. Younger individuals had higher 25(OH)D concentrations (Table 3). For this reason, in the multiple regression vitamin D status analysis, we chose to use age as the predictive variable, adjusted by gender and ethnicity. Among the other parameters evaluated in the model, ionized calcium, PTH and month of the year, which are other variables that present known correlation with 25(OH)D, maintained significance [16,17].

It is known that the 25(OH)D plasma concentrations follow a seasonal variation, demonstrated in places of more temperate weather [16,18,19]. Our data clearly demonstrate the existence of a seasonal pattern for 25(OH)D concentrations throughout the year that follows the ultraviolet radiation distribution and does not change over time. This phenomenon was evident in all the groups, except for the NH one (Table 4). In this group, although the group age was similar to the one in CD, the mean of the values in summer was not higher than that obtained in winter. This suggests that sunlight exposure was not enough for this group. On the other hand, it was observed that the subjects from CD reached values 34% higher in the summer, almost reaching the increment rate observed for young people, which was 47%.

Gannagé-Yared *et al.* demonstrated that there are influences from life habits on 25(OH)D concentration (in this study the use of veil was evaluated) [20]. Other authors demonstrated that the seasonal variation also exerts influences on bone remodeling markers, bone mass and fractures [19,21]. In the same way, the individuals from PA group demonstrated a well defined seasonal variation when prospectively evaluated, except for the ones older than 71 years old who presented a pattern similar to that of the NH and CD groups [9]. An interesting fact is that the group PA had some sunlight exposure during their physical activities at least twice a week for 1 h (in this group the use of sunscreen was evaluated and no significant variation was found between users and non-users). These data suggest that if more frequently exposed to sunlight, the individuals younger than 70 years old can achieve an adequate vitamin D status [22-24]. This is probably one of the advantages of living in lower latitudes, where sunlight is more abundant.

In our study, it was possible to observe the correlation that exists between PTH and 25(OH)D, which was expressed through an exponential equation (Figure 1), the same way it has been described [20,25,26]. The concentration from which the correlation between 25(OH)D and PTH was lost was > 75.0 nmol/L (Table 2), a value similar to previous reports [27]. It means that above this concentration, sufficiency of vitamin D does not interfere with PTH secretion. We observed that the lower concentrations of 25(OH)D are associated with secondary hyperparathyroidism and that was more frequent in NH and CD (Tables 1, 4 and 3). Among the young individuals evaluated in this study, we found a higher 25(OH)D mean. However, for a good proportion of the samples the values were below the sufficiency limit, but besides that, no correlation with PTH was observed (r = −0.018, p = 0.876). Among young individuals PTH does not seem to be a good parameter to indirectly evaluate vitamin D status [10].

For the multiple regression analysis, setting PTH level as the depending variable and age as the adjusted predictive variable, the evaluated parameters that maintained the significance in the model were 25(OH)D, creatinine and ionized calcium, variables which are known to exert influence on PTH levels [28,29]. Table 3 suggested that PTH levels increased with age, possibly due to several factors acting together, such as vitamin D insufficiency/deficiency, renal function decrease (1α-hydroxylation) and the influence of prescription drugs on vitamin D metabolism [29,30]. Another important factor is the decreased ingestion and intestinal absorption of calcium [31-33].

The primary objective of hypovitaminosis D correction is promoting the maximum intestinal calcium absorption, reducing the frequency of falls and correcting the secondary hyperparathyroidism [27]. According to Bischoff-Ferrari *et al.*, the supplementation with at least 700 IU of vitamin D/d was enough to reduce the risk of vertebral and non-vertebral fractures [34]. Besides that, there are other non-skeletal effects which have been attributed to vitamin D status, such as the prevention of cancer and autoimmune diseases [4]. In spite of all this knowledge, the number of individuals who received vitamin D supplements was very low. When the mean 25(OH)D concentration was evaluated for this group, we observed that although it was higher than the mean value for individuals who did not receive supplementation, it was still below the ideal limit (73% of these individuals presented concentrations < 75.0 nmol/L) [3,4]. Therefore the vitamin D doses these individuals received (200–400 IU/d) were not enough for the prevention of hypovitaminosis D. The Endocrine Practice Guidelines recommends adults need 1,500-2,000 IU vitamin D/d to prevent vitamin D deficiency [35].

These results point out to the need for governmental public health action, which could implement measures such as vitamin D supplement distribution to nursing homes, food supplementation and medical education about the high prevalence of this condition among the elderly, considering the associated risk for fractures.

Positive aspects of our paper include that we evaluated a large number of adults from different populations collected in the same city and analyzed by the same method. It was possible to demonstrate that hypovitaminosis D is a public health problem even in Brazil. However what is still lacking is the relationship with vitamin D status with the amount of sun exposure.

Due to the high degree of miscegenation of the Brazilian population, it is always difficult to classify them ethnically. Nevertheless, most of the evaluated individuals were white (84.4%). According to the 2000 Brazilian Demographic Census for the city of São Paulo, the white population is the majority (67.0% of population, data from IBGE- Instituto Brasileiro de Geografia e Estatística). However, our data cannot be extrapolated to all different regions of the country, because, besides different latitudes and weather conditions, the population presents different genetic backgrounds and habits, which are important factors for the serum vitamin D status.

In addition, several other factors potentially affect vitamin D status such as genetic factors, adiposity, dietary intake, intestinal malabsorption and impaired hydroxylation in the liver and kidneys.

## Conclusions

We found very low 25(OH)D concentrations for the elderly when compared to younger people. This finding is possibly due to age and habit-related sunlight exposure differences. The existence of a seasonal variation over 25(OH)D concentrations is evident, as well as the influence of age is. Secondary hyperparathyroidism was frequent among the older individuals and was associated with 25(OH)D levels, creatinine and ionized calcium. According to our data, PTH values tend to plateau above 75.0 nmol/L. We found that 73.3% of the sample population studied is below this value.

## Abbreviations

UNIFESP: Universidade Federal de São Paulo (Federal University of São Paulo); SPADES: São PAulo Vitamin DEvaluation Study; 25(OH)D: 25 hidroxyvitamin D; NH: Nursing homes; CD: Community dwellings; PA: Physical activity; PTH: Parathyroid hormone; BMI: Body mass index; UVB: Ultraviolet radiation B; IBGE: Instituto Brasileiro de Geografia e Estatística (Brazilian Institute of Geography and Statistics).

## Competing interests

The authors declare that they have no competing interests.

## Authors’ contributions

SSM - main author. GLS – provided the data of NH and CD groups, participated in its design. ISK - carried out the immunoassays. LFH - carried out the immunoassays. MSC - provided the data of NH and CD groups, participated in its design. LRR - provided the data of NH and CD groups, participated in its design. MLC – conceived of the study, and participated in its design and coordination and helped to draft the manuscript. All authors read and approved the final manuscript.

## Authors’ information

SSM – M.D., Ph.D. at the UNIFESP (Bone Metabolism section), and Professor at the Santa Casa de São Paulo Medical School

MLC – M.D., Ph.D. and Professor at the UNIFESP (Bone Metabolism section)

## Pre-publication history

The pre-publication history for this paper can be accessed here:

http://www.biomedcentral.com/1472-6823/13/14/prepub
